# Study of HCP (Hexagonal Close-Packed) Crystal Structure Lattice through Topological Descriptors

**DOI:** 10.1155/2022/6069666

**Published:** 2022-07-31

**Authors:** Guoping Zhang, Saadia Saeed, Adnan Aslam, Salma Kanwal, Nazeran Idrees, Tahira Sumbal Shaikh

**Affiliations:** ^1^School of Software, Pingdingshan University, Pingdingshan, Henan 467000, China; ^2^Henan International Joint Laboratory for Multidimensional Topology and Carcinogenic Charateristics Analysis of Atmospheric Particulate Matter PM2.5, Pingdingshan, Henan 467000, China; ^3^Department of Mathematics, Lahore College for Women University, Lahore, Pakistan; ^4^University of Engineering and Technology, Lahore (RCET) 54000, Lahore, Pakistan; ^5^Department of Mathematics, Government College University, Faisalabad, Pakistan

## Abstract

Chemical graph theory is a multidisciplinary field where the structure of the molecule is analyzed as a graphical structure. Chemical descriptors are one of the most important ideas employed in chemical graph theory; this is to associate a numerical value with a graph structure that often has correlation with corresponding chemical properties. In this paper, we investigate another very important closed-packed usual crystal structure defined as HCP (Hexagonal Close-Packed) crystal structure and its lattice formed by arranging its unit cells in a dimension for topological descriptors based on a neighborhood degree, reverse degree, and degree. Furthermore, we classify which descriptor is more dominating.

## 1. Introduction

Chemical graph theory plays a considerate role to investigate a wide range of inorganic and organic chemical structures by studying them through graphical representation. A topological descriptor is a mathematical entity based on certain topological features of chemical structure which correlates with corresponding chemical properties [1–7]. In this research work, we study the most symmetrical and complex close-packed structure defined as HCP(*n*) using two-dimensional topological descriptors. In graph theory [8], degree of a vertex *s* in a corresponding graph is the number of edges incident with that vertex defined as *d*_*s*_. Reverse degree of a vertex *s* is defined as ℝ_*s*_ = Δ(*G*) + 1 − *d*_*s*_, where Δ(*G*) is the maximum degree of a vertex in a graph *G*. Neighborhood degree of a vertex *s* in *G* is defined as *δ*_*s*_ = ∑_*t*∈*N*_*s*__*d*_*t*_, where *N*_*S*_ represents the neighborhood degree of a vertex *s*.

**Figure 1:**
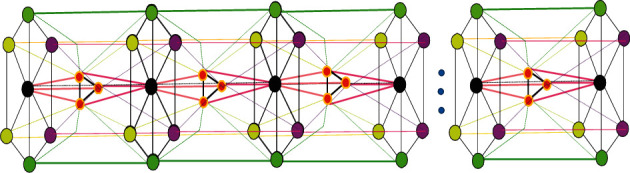
HCP(*n*); hexagonal close packing lattice formed by *n* unit cells.

Hexagonal Close Packing (**HCP**) consists of alternating layers of spheres of atoms (vertices) arranged in a hexagon, with one additional atom (vertex) at the center as shown in Figure 1. Another layer of atoms is sandwiched between these two hexagonal layers which are triangular (three atoms form a triangle by three edges), and the atoms (vertices) of this layer fill the tetrahedral holes created by the top and bottom [9]. The edge set *E*(*G*) of HCP contains the edges that connect the atoms that are nearest to each other by an edge. In this context, we represent edges by both a filled and a dotted line to clarify the bonding between two atoms in a 3D HCP(*n*). The middle layer atoms also share a bond with both hexagonal layers by following a symmetrical pattern.

Elements that form an HCP crystal structure are zirconium, ruthenium, hafnium, and many more. It is the most strong and brittle structure that is highly found in metals that do not have a very smooth symmetry as in cubic structures, but it contains a vast and stronger metallic property than a usual hexagonal crystal structure.

## 2. Preliminaries

We give a brief view of some well-known TIs for which we compute closed formulas corresponding to each crystal structure.

Every index is defined as

(1) $$\begin{array}{c} TI\left(G\right)=\sum_{\text{edges}}f\left(H,K\right). \end{array}$$

In Table 1, we give a brief view for each descriptor in terms of its function defining in above equation as *f*(*H*, *K*).HCP(*n*) is a very densely symmetric crystal structure with a complex bonding between its atoms. As it is not a tough thing to study any planar chem structure via descriptors but, here our goal is to perform evaluation for not a planar but a 3-dimensional structure with a complex symmetry in comparison to cubic structure. We regard the study for the work done to date for complex and 3D crystals, including diamond crystal structures [23], FCC [24], and BCC [25] crystal unit cells that have been investigated by utilizing the definitions from chemical graph theory [5], and allows to study organic chemistry, cordially here, metals and minerals attaining crystal unit cells.

## 3. Formulation

In this section, we consider that a graph *G* is HCP(*n*) defined as hexagonal close-packed crystal structure lattice consisting of *n* unit cells arranged in one dimension. *V*(HCP(*n*)) is defined as vertex set of hexagonal close-packed lattice, and edge set is defined as *E*(HCP(*n*)) where for *n* ordered lattice *V*(HCP(n))=10*n*+7, *E*(HCP(*n*))=40*n*+12.

### 3.1. Degree Based

We utilize Table 2 to compute closed forms for the defined descriptors dependent on degree.

(1) First Zagreb index of **HCP**(**n**) is defined as follows:
  (2) $$\begin{array}{c} M_{1}\left(G\right)=\sum_{s\in V\left(G\right)}\left(d_{s}+d_{t}\right)=12\left(10\right)+12\left(15\right)+12\left(13\right)+3n\left(16\right)+\\ 6\left(18\right)+\left(12n-18\right)\left(14\right)+\left(12n-12\right)\left(15\right)\\ +\left(6n-6\right)\left(21\right)+2\left(24\right)+\left(n-2\right)\left(28\right)+\\ \left(6n-6\right)\left(22\right)+12\left(12\right)=634n-10. \end{array}$$
(2) Second Zagreb index of **HCP**(**n**) is defined as follows:
  (3) $$\begin{array}{c} M_{2}\left(G\right)=\sum_{st\in E\left(G\right)}d_{s}d_{t}=12\left(25\right)+12\left(50\right)+12\left(40\right)+3n\left(64\right)+6\left(80\right)\\ +\left(12n-18\right)\left(49\right)+\left(12n-12\right)\left(56\right)+\left(6n-6\right)\left(98\right)\\ +2\left(140\right)+\left(n-2\right)\left(196\right)+\left(6n-6\right)\left(112\right)\\ +12\left(35\right)=2908n-646. \end{array}$$
(3) General Randić index of **HCP**(**n**) is defined as follows:
  (4) $$\begin{array}{c} R_{\alpha }\left(G\right)=\sum_{st\in E\left(G\right)}{\left(d_{s}d_{t}\right)}^{\alpha }=12{\left(d_{s}d_{t}\right)}^{\alpha }+12{\left(d_{s}d_{t}\right)}^{\alpha }+12{\left(d_{s}d_{t}\right)}^{\alpha }\\ +3n{\left(d_{s}d_{t}\right)}^{\alpha }+6{\left(d_{s}d_{t}\right)}^{\alpha }+\\ \left(12n-18\right){\left(d_{s}d_{t}\right)}^{\alpha }+\left(12n-12\right){\left(d_{s}d_{t}\right)}^{\alpha }+\\ \left(6n-6\right){\left(d_{s}d_{t}\right)}^{\alpha }+2{\left(d_{s}d_{t}\right)}^{\alpha }+\left(n-2\right){\left(d_{s}d_{t}\right)}^{\alpha }+\\ \left(6n-6\right){\left(d_{s}d_{t}\right)}^{\alpha }+12{\left(d_{s}d_{t}\right)}^{\alpha }. \end{array}$$
  (5) $$\begin{array}{c} R_{1}\left(G\right)=\sum_{st\in E\left(G\right)}{\left(d_{s}d_{t}\right)}^{1}=12{\left(25\right)}^{1}+12{\left(50\right)}^{1}\\ +12{\left(40\right)}^{1}+3n{\left(64\right)}^{1}+6{\left(80\right)}^{1}+\left(12n-18\right){\left(49\right)}^{1}\\ +\left(12n-12\right){\left(56\right)}^{1}+\left(6n-6\right){\left(98\right)}^{1}+2{\left(140\right)}^{1}\\ +\left(n-2\right){\left(196\right)}^{1}+\left(6n-6\right){\left(112\right)}^{1}+12{\left(35\right)}^{1}=M_{2}\left(G\right).\\ 12{\left(50\right)}^{-1}+12{\left(40\right)}^{-1}+3n{\left(64\right)}^{-1}+\\ 6{\left(80\right)}^{-1}+\left(12n-18\right){\left(49\right)}^{-1}+\left(12n-12\right){\left(56\right)}^{-1}+\left(6n-6\right){\left(98\right)}^{-1}+2{\left(140\right)}^{-1}\\ +\left(n-2\right){\left(196\right)}^{-1}+\left(6n-6\right){\left(112\right)}^{-1}\\ +12{\left(35\right)}^{-1}=0.7455102041+0.5790816327n.\\ 12{\left(50\right)}^{\frac{1}{2}}+12{\left(40\right)}^{\frac{1}{2}}+3n{\left(64\right)}^{\frac{1}{2}}+6{\left(80\right)}^{\frac{1}{2}}+\\ \left(12n-18\right){\left(49\right)}^{\frac{1}{2}}+\left(12n-12\right){\left(56\right)}^{\frac{1}{2}}+\left(6n-6\right){\left(98\right)}^{\frac{1}{2}}+\\ 2{\left(140\right)}^{\frac{1}{2}}+\left(n-2\right){\left(196\right)}^{\frac{1}{2}}\\ +\left(6n-6\right){\left(112\right)}^{\frac{1}{2}}+12{\left(35\right)}^{\frac{1}{2}}=2.375607208+334.6947784n.\\ +12{\left(40\right)}^{-\frac{1}{2}}+3n{\left(64\right)}^{-\frac{1}{2}}+6{\left(80\right)}^{-\frac{1}{2}}+\\ \left(12n-18\right){\left(49\right)}^{-\frac{1}{2}}+\left(12n-12\right){\left(56\right)}^{-\frac{1}{2}}+\left(6n-6\right){\left(98\right)}^{-\frac{1}{2}}+\\ 2{\left(140\right)}^{-\frac{1}{2}}+\left(n-2\right){\left(196\right)}^{-\frac{1}{2}}+\left(6n-6\right){\left(112\right)}^{-\frac{1}{2}}+12{\left(35\right)}^{-\frac{1}{2}}\\ =3.371752924+4.937319973n. \end{array}$$
  (6) $$\begin{array}{c} R_{-1}\left(G\right)=\sum_{st\in E\left(G\right)}{\left(d_{s}d_{t}\right)}^{-1}=12{\left(25\right)}^{-1}+\\ 12{\left(50\right)}^{-1}+12{\left(40\right)}^{-1}+3n{\left(64\right)}^{-1}+\\ 6{\left(80\right)}^{-1}+\left(12n-18\right){\left(49\right)}^{-1}+\left(12n-12\right){\left(56\right)}^{-1}+\left(6n-6\right){\left(98\right)}^{-1}+2{\left(140\right)}^{-1}\\ +\left(n-2\right){\left(196\right)}^{-1}+\left(6n-6\right){\left(112\right)}^{-1}\\ +12{\left(35\right)}^{-1}=0.7455102041+0.5790816327n.\\ 12{\left(50\right)}^{\frac{1}{2}}+12{\left(40\right)}^{\frac{1}{2}}+3n{\left(64\right)}^{\frac{1}{2}}+6{\left(80\right)}^{\frac{1}{2}}+\\ \left(12n-18\right){\left(49\right)}^{\frac{1}{2}}+\left(12n-12\right){\left(56\right)}^{\frac{1}{2}}+\left(6n-6\right){\left(98\right)}^{\frac{1}{2}}+\\ 2{\left(140\right)}^{\frac{1}{2}}+\left(n-2\right){\left(196\right)}^{\frac{1}{2}}\\ +\left(6n-6\right){\left(112\right)}^{\frac{1}{2}}+12{\left(35\right)}^{\frac{1}{2}}=2.375607208+334.6947784n.\\ +12{\left(40\right)}^{-\frac{1}{2}}+3n{\left(64\right)}^{-\frac{1}{2}}+6{\left(80\right)}^{-\frac{1}{2}}+\\ \left(12n-18\right){\left(49\right)}^{-\frac{1}{2}}+\left(12n-12\right){\left(56\right)}^{-\frac{1}{2}}+\left(6n-6\right){\left(98\right)}^{-\frac{1}{2}}+\\ 2{\left(140\right)}^{-\frac{1}{2}}+\left(n-2\right){\left(196\right)}^{-\frac{1}{2}}+\left(6n-6\right){\left(112\right)}^{-\frac{1}{2}}+12{\left(35\right)}^{-\frac{1}{2}}\\ =3.371752924+4.937319973n. \end{array}$$
  (7) $$\begin{array}{c} R_{\frac{1}{2}}\left(G\right)=\sum_{st\in E\left(G\right)}{\left(d_{s}d_{t}\right)}^{\frac{1}{2}}=12{\left(25\right)}^{\frac{1}{2}}+\\ 12{\left(50\right)}^{\frac{1}{2}}+12{\left(40\right)}^{\frac{1}{2}}+3n{\left(64\right)}^{\frac{1}{2}}+6{\left(80\right)}^{\frac{1}{2}}+\\ \left(12n-18\right){\left(49\right)}^{\frac{1}{2}}+\left(12n-12\right){\left(56\right)}^{\frac{1}{2}}+\left(6n-6\right){\left(98\right)}^{\frac{1}{2}}+\\ 2{\left(140\right)}^{\frac{1}{2}}+\left(n-2\right){\left(196\right)}^{\frac{1}{2}}\\ +\left(6n-6\right){\left(112\right)}^{\frac{1}{2}}+12{\left(35\right)}^{\frac{1}{2}}=2.375607208+334.6947784n.\\ +12{\left(40\right)}^{-\frac{1}{2}}+3n{\left(64\right)}^{-\frac{1}{2}}+6{\left(80\right)}^{-\frac{1}{2}}+\\ \left(12n-18\right){\left(49\right)}^{-\frac{1}{2}}+\left(12n-12\right){\left(56\right)}^{-\frac{1}{2}}+\left(6n-6\right){\left(98\right)}^{-\frac{1}{2}}+\\ 2{\left(140\right)}^{-\frac{1}{2}}+\left(n-2\right){\left(196\right)}^{-\frac{1}{2}}+\left(6n-6\right){\left(112\right)}^{-\frac{1}{2}}+12{\left(35\right)}^{-\frac{1}{2}}\\ =3.371752924+4.937319973n. \end{array}$$
  (8) $$\begin{array}{c} R_{-\frac{1}{2}}\left(G\right)=\sum_{st\in E\left(G\right)}{\left(d_{s}d_{t}\right)}^{-\frac{1}{2}}=12{\left(25\right)}^{-\frac{1}{2}}+12{\left(50\right)}^{-\frac{1}{2}}\\ +12{\left(40\right)}^{-\frac{1}{2}}+3n{\left(64\right)}^{-\frac{1}{2}}+6{\left(80\right)}^{-\frac{1}{2}}+\\ \left(12n-18\right){\left(49\right)}^{-\frac{1}{2}}+\left(12n-12\right){\left(56\right)}^{-\frac{1}{2}}+\left(6n-6\right){\left(98\right)}^{-\frac{1}{2}}+\\ 2{\left(140\right)}^{-\frac{1}{2}}+\left(n-2\right){\left(196\right)}^{-\frac{1}{2}}+\left(6n-6\right){\left(112\right)}^{-\frac{1}{2}}+12{\left(35\right)}^{-\frac{1}{2}}\\ =3.371752924+4.937319973n. \end{array}$$
(3a) When *α*=1,
(3b) When *α*=−1,
(3c) When *α*=1/2,
(3d) When *α*=−1/2,
(4) Atom bond connectivity index of **HCP**(**n**) is defined as follows:
  (9) $$\begin{array}{c} ABC\left(G\right)=\sum_{st\in E\left(G\right)}\sqrt{\frac{d_{s}+d_{t}-2}{d_{s}d_{t}}}=12\left(\sqrt{\frac{8}{25}}\right)\\ +12\left(\sqrt{\frac{13}{50}}\right)+12\left(\sqrt{\frac{11}{40}}\right)+3n\left(\sqrt{\frac{7}{32}}\right)\\ +6\left(\sqrt{\frac{1}{5}}\right)+\left(12n-18\right)\left(\sqrt{\frac{12}{49}}\right)+\left(12n-12\right)\left(\sqrt{\frac{13}{56}}\right)\\ +\left(6n-6\right)\left(\sqrt{\frac{19}{98}}\right)+2\left(\sqrt{\frac{11}{70}}\right)+\left(n-2\right)\left(\sqrt{\frac{13}{98}}\right)+\left(6n-6\right)\left(\sqrt{\frac{5}{28}}\right)\\ +12\left(\sqrt{\frac{2}{7}}\right)=8.495057575+18.66489626n. \end{array}$$
(5) The geometric arithmetic index of **HCP**(**n**) is defined as follows:
  (10) $$\begin{array}{c} GA\left(G\right)=\sum_{st\in E\left(G\right)}\frac{2\sqrt{d_{s}d_{t}}}{d_{s}+d_{t}}=12\left(\frac{2\sqrt{25}}{10}\right)+12\left(\frac{2\sqrt{50}}{15}\right)\\ +12\left(\frac{2\sqrt{40}}{13}\right)+3n\left(\frac{2\sqrt{64}}{16}\right)+6\left(\frac{2\sqrt{80}}{18}\right)+\left(12n-18\right)\left(\frac{2\sqrt{49}}{14}\right)\\ +\left(12n-12\right)\left(\frac{2\sqrt{56}}{15}\right)+\left(6n-6\right)\left(\frac{2\sqrt{98}}{21}\right)+2\left(\frac{2\sqrt{140}}{24}\right)\\ +\left(n-2\right)\left(\frac{2\sqrt{196}}{28}\right)+\left(6n-6\right)\left(\frac{2\sqrt{112}}{22}\right)\\ +12\left(\frac{2\sqrt{35}}{12}\right)=39.4027062n+11.35413853. \end{array}$$
(6) Exponential Reduced Zagreb index of **HCP**(**n**) is defined as follows:
  (11) $$\begin{array}{c} e^{\mathrm{RM}2}\left(G\right)=\sum_{\mathrm{st}\in E\left(G\right)}e^{\left(d_{s}-1\right)\left(d_{t}-1\right)}=12\left(e^{\left(16\right)}\right)+12\left(e^{\left(36\right)}\right)\\ +12\left(e^{\left(28\right)}\right)+3n\left(e^{\left(49\right)}\right)+6\left(e^{\left(63\right)}\right)\\ +\left(12n-18\right)\left(e^{\left(36\right)}\right)+\left(12n-12\right)\left(e^{\left(42\right)}\right)+\left(6n-6\right)\left(e^{\left(78\right)}\right)\\ +2\left(e^{\left(117\right)}\right)+\left(n-2\right)\left(e^{\left(169\right)}\right)+\left(6n-6\right)\left(e^{\left(91\right)}\right)+12\left(e^{\left(24\right)}\right)\\ =2.487524928\times 10^{73}n-4.975049857\times 10^{73}. \end{array}$$

### 3.2. Reverse Degree Based

We utilize Table 3 to compute closed forms for the defined descriptors dependent on reverse degree of a vertex set of HCP(*n*). where maximum degree for HCP(*n*) = Δ(HCP(*n*) = 14, which we use to compute reverse degree for the entire vertex set of lattice.

(1) Reverse Randić index of **HCP**(**n**) is as follows where *α* = ±1, ±1/2:
  (1a) When *α* = 1,
    (12) $$\begin{array}{c} \mathbb{R}R_{1}\left(G\right)=\sum_{st\in E\left(G\right)}{\left[{\mathbb{R}}_{s}{\mathbb{R}}_{t}\right]}^{1}=\sum_{st\in E_{1}}\left[{\mathbb{R}}_{s}{\mathbb{R}}_{t}\right]+\sum_{st\in E_{2}}\left[{\mathbb{R}}_{s}{\mathbb{R}}_{t}\right]+\cdots +\sum_{st\in E_{12}}\left[{\mathbb{R}}_{s}{\mathbb{R}}_{t}\right]\\ =12\left(10\times 10\right)+12\left(10\times 5\right)+12\left(10\times 7\right)+3n\left(7\times 7\right)+6\left(7\times 5\right)\\ +\left(12n-18\right)\left(8\times 8\right)+\left(12n-12\right)\left(8\times 7\right)+\left(6n-6\right)\left(8\times 1\right)+2\left(5\times 1\right)\\ +\left(n-2\right)\left(1\times 1\right)+\left(6n-6\right)\left(7\times 1\right)+12\left(10\times 8\right)\\ =1904+1678n. \end{array}$$
  (1b) When *α* = −1,
    (13) $$\begin{array}{c} \mathbb{R}R_{-1}\left(G\right)=\sum_{st\in E\left(G\right)}{\left[{\mathbb{R}}_{s}{\mathbb{R}}_{t}\right]}^{-1}=\sum_{st\in E_{1}}{\left[{\mathbb{R}}_{s}{\mathbb{R}}_{t}\right]}^{-1}+\sum_{st\in E_{2}}{\left[{\mathbb{R}}_{s}{\mathbb{R}}_{t}\right]}^{-1}+\cdots +\sum_{st\in E_{12}}{\left[{\mathbb{R}}_{s}{\mathbb{R}}_{t}\right]}^{-1}\\ =12{\left(10\times 10\right)}^{-1}+12{\left(10\times 5\right)}^{-1}+12{\left(10\times 7\right)}^{-1}+3n{\left(7\times 7\right)}^{-1}\\ +6{\left(7\times 5\right)}^{-1}+\left(12n-18\right){\left(8\times 8\right)}^{-1}+\left(12n-12\right){\left(8\times 7\right)}^{-1}+\left(6n-6\right){\left(8\times 1\right)}^{-1}\\ +2{\left(5\times 1\right)}^{-1}+\left(n-2\right){\left(1\times 1\right)}^{-1}+\left(6n-6\right){\left(7\times 1\right)}^{-1}+12{\left(10\times 8\right)}^{-1}\\ =\frac{2407n}{784}-\frac{15959}{5600}. \end{array}$$
  (1c) When *α* = 1/2,
    (14) $$\begin{array}{c} \mathbb{R}R_{\frac{1}{2}}\left(G\right)=\sum_{st\in E\left(G\right)}{\left[{\mathbb{R}}_{s}{\mathbb{R}}_{t}\right]}^{\frac{1}{2}}=\sum_{st\in E_{1}}{\left[{\mathbb{R}}_{s}{ℜ}_{t}\right]}^{\frac{1}{2}}+\sum_{st\in E_{2}}{\left[{\mathbb{R}}_{s}{\mathbb{R}}_{t}\right]}^{\frac{1}{2}}+\cdots +\sum_{st\in E_{12}}{\left[{\mathbb{R}}_{s}{\mathbb{R}}_{t}\right]}^{\frac{1}{2}}\\ =12{\left(10\times 10\right)}^{\frac{1}{2}}+12{\left(10\times 5\right)}^{\frac{1}{2}}+12{\left(10\times 7\right)}^{\frac{1}{2}}+3n{\left(7\times 7\right)}^{\frac{1}{2}}+6{\left(7\times 5\right)}^{\frac{1}{2}}\\ +\left(12n-18\right){\left(8\times 8\right)}^{\frac{1}{2}}+\left(12n-12\right){\left(8\times 7\right)}^{\frac{1}{2}}+\left(6n-6\right){\left(8\times 1\right)}^{\frac{1}{2}}+2{\left(5\times 1\right)}^{\frac{1}{2}}\\ +\left(n-2\right){\left(1\times 1\right)}^{\frac{1}{2}}+\left(6n-6\right){\left(7\times 1\right)}^{\frac{1}{2}}+12{\left(10\times 8\right)}^{\frac{1}{2}}\\ =240.6448479n+183.9070466. \end{array}$$
  (1d) When *α* = −1/2,
    (15) $$\begin{array}{c} \mathbb{R}R_{-\frac{1}{2}}\left(G\right)=\sum_{st\in E\left(G\right)}{\left[{\mathbb{R}}_{s}{\mathbb{R}}_{t}\right]}^{-\frac{1}{2}}=\sum_{st\in E_{1}}{\left[{\mathbb{R}}_{s}{\mathbb{R}}_{t}\right]}^{\frac{-1}{2}}+\sum_{st\in E_{2}}{\left[{\mathbb{R}}_{s}{\mathbb{R}}_{t}\right]}^{-\frac{1}{2}}+\cdots +\sum_{st\in E_{12}}{\left[{\mathbb{R}}_{s}{\mathbb{R}}_{t}\right]}^{-\frac{1}{2}}\\ =12{\left(10\times 10\right)}^{-\frac{1}{2}}+12{\left(10\times 5\right)}^{-\frac{1}{2}}+12{\left(10\times 7\right)}^{-\frac{1}{2}}+3n{\left(7\times 7\right)}^{-\frac{1}{2}}+6{\left(7\times 5\right)}^{-\frac{1}{2}}+\\ \left(12n-18\right){\left(8\times 8\right)}^{-\frac{1}{2}}+\left(12n-12\right){\left(8\times 7\right)}^{-\frac{1}{2}}+\left(6n-6\right){\left(8\times 1\right)}^{-\frac{1}{2}}+2{\left(5\times 1\right)}^{-\frac{1}{2}}+\left(n-2\right){\left(1\times 1\right)}^{-\frac{1}{2}}\\ +\left(6n-6\right){\left(7\times 1\right)}^{-\frac{1}{2}}+12{\left(10\times 8\right)}^{-\frac{1}{2}}\\ =8.921246062n-3.503847478. \end{array}$$
(2) Reverse atom bond connectivity index of **HCP**(**n**) is defined as follows:
  (16) $$\begin{array}{c} \mathbb{R}ABC\left(G\right)=\sum_{st\in E_{1}}\sqrt{\frac{{\mathbb{R}}_{s}+{\mathbb{R}}_{t}-2}{{\mathbb{R}}_{s}{\mathbb{R}}_{t}}}=\sum_{st\in E_{2}}\sqrt{\frac{{\mathbb{R}}_{s}+{\mathbb{R}}_{t}-2}{{\mathbb{R}}_{s}{\mathbb{R}}_{t}}}+\sum_{st\in E_{3}}\sqrt{\frac{{\mathbb{R}}_{s}+{\mathbb{R}}_{t}-2}{{\mathbb{R}}_{s}{\mathbb{R}}_{t}}}+\cdots +\sum_{st\in E_{12}}\sqrt{\frac{{\mathbb{R}}_{s}+{\mathbb{R}}_{t}-2}{{\mathbb{R}}_{s}{\mathbb{R}}_{t}}}\\ =12\left(\sqrt{\frac{10+10-2}{10\times 10}}\right)+12\left(\sqrt{\frac{10+5-2}{10\times 5}}\right)+12\left(\sqrt{\frac{10+7-2}{10\times 7}}\right)\\ +\left(3n\right)\left(\sqrt{\frac{7+7-2}{7\times 7}}\right)+6\left(\sqrt{\frac{7+5-2}{7\times 5}}\right)+\left(12n-18\right)\left(\sqrt{\frac{8+8-2}{8\times 8}}\right)\\ +\left(12n-12\right)\left(\sqrt{\frac{8+7-2}{8\times 7}}\right)+\left(6n-6\right)\left(\sqrt{\frac{8+1-2}{8\times 1}}\right)+2\left(\sqrt{\frac{5+1-2}{5\times 1}}\right)\\ +\left(n-2\right)\left(\sqrt{\frac{1+1-2}{1\times 1}}\right)+\left(6n-6\right)\left(\sqrt{\frac{7+1-2}{7\times 2}}\right)+12\left(\sqrt{\frac{10+8-2}{108}}\right)\\ =24.04625241n+1.759584801. \end{array}$$
(3) Reverse geometric arithmetic index of **HCP**(**n**) is defined as follows:
  (17) $$\begin{array}{c} \mathbb{R}GA\left(G\right)=\sum_{st\in E\left(G\right)}\frac{2\sqrt{{\mathbb{R}}_{s}{\mathbb{R}}_{t}}}{{\mathbb{R}}_{s}+{\mathbb{R}}_{t}}=\sum_{st\in E_{1}}\frac{2\sqrt{{\mathbb{R}}_{s}{\mathbb{R}}_{t}}}{{\mathbb{R}}_{s}+{\mathbb{R}}_{t}}+\sum_{st\in E_{2}}\frac{2\sqrt{{\mathbb{R}}_{s}{\mathbb{R}}_{t}}}{{\mathbb{R}}_{s}+{\mathbb{R}}_{t}}+\sum_{st\in E_{3}}\frac{2\sqrt{{\mathbb{R}}_{s}{\mathbb{R}}_{t}}}{{\mathbb{R}}_{s}+{\mathbb{R}}_{t}}+\cdots +\sum_{st\in E_{12}}\frac{2\sqrt{{\mathbb{R}}_{s}{\mathbb{R}}_{t}}}{{\mathbb{R}}_{s}+{\mathbb{R}}_{t}}\\ =12\left(\frac{2\sqrt{10\times 10}}{10+10}\right)+12\left(\frac{2\sqrt{10\times 5}}{10+5}\right)+12\left(\frac{2\sqrt{10\times 7}}{10+7}\right)+3n\left(\frac{2\sqrt{7\times 7}}{7+7}\right)+6\left(\frac{2\sqrt{7\times 5}}{7+5}\right)\\ +\left(12n-18\right)\left(\frac{2\sqrt{8\times 8}}{8+8}\right)+\left(12n-12\right)\left(\frac{2\sqrt{8\times 7}}{8+7}\right)+\left(6n-6\right)\left(\frac{2\sqrt{8\times 1}}{8+1}\right)+2\left(\frac{2\sqrt{5\times 1}}{5+1}\right)\\ +\left(n-2\right)\left(\frac{2\sqrt{1\times 1}}{1+1}\right)+\left(6n-6\right)\left(\frac{2\sqrt{7\times 1}}{7+1}\right)\\ +12\left(\frac{2\sqrt{10\times 8}}{10+8}\right)=35.71316677n+14.74470034. \end{array}$$
(4) Reverse hyper Zagreb index of **HCP**(**n**) is defined as follows:
  (18) $$\begin{array}{c} \mathbb{R}HM\left(G\right)=\sum_{st\in E\left(G\right)}{\left({\mathbb{R}}_{s}+{\mathbb{R}}_{t}\right)}^{2}=\sum_{st\in E_{1}}{\left({\mathbb{R}}_{s}+{\mathbb{R}}_{t}\right)}^{2}+\sum_{st\in E_{2}}{\left({\mathbb{R}}_{s}+{\mathbb{R}}_{t}\right)}^{2}+\sum_{st\in E_{3}}{\left({\mathbb{R}}_{s}+{\mathbb{R}}_{t}\right)}^{2}\\ +\cdots +\sum_{st\in E_{12}}{\left({\mathbb{R}}_{s}+{\mathbb{R}}_{t}\right)}^{2}=12{\left(10+10\right)}^{2}+12{\left(10+5\right)}^{2}+12{\left(10+7\right)}^{2}\\ +3n{\left(7+7\right)}^{2}+6{\left(7+5\right)}^{2}\\ +\left(12n-18\right){\left(8+8\right)}^{2}+\left(12n-12\right){\left(8+7\right)}^{2}\\ +\left(6n-6\right){\left(8+1\right)}^{2}+2{\left(5+1\right)}^{2}\\ +\left(n-2\right){\left(1+1\right)}^{2}+\left(6n-6\right){\left(7+1\right)}^{2}+12{\left(10+8\right)}^{2}\\ =7234n+7606. \end{array}$$

### 3.3. Neighborhood Degree Based

We utilize Table 4 to evaluate closed formulas for the neighborhood degree-based descriptors representing topological properties.

(1) Neighborhood version of first Zagreb index of **HCP**(**n**) is defined as follows:
  (19) $$\begin{array}{c} M_{1}^{*}\left(G\right)=\sum_{st\in E\left(G\right)}\left(\delta_{s}+\delta_{t}\right)=\sum_{st\in E_{1}}\left(\delta_{s}+\delta_{t}\right)+\sum_{st\in E_{2}}\left(\delta_{s}+\delta_{t}\right)+\sum_{st\in E_{3}}\left(\delta_{s}+\delta_{t}\right)+\cdots \\ +\sum_{st\in E_{21}}\left(\delta_{s}+\delta_{t}\right)=12\left(70\right)+12\left(103\right)+12\left(99\right)+12\left(91\right)+2\left(182\right)\\ +12\left(112\right)+12\left(120\right)+6\left(178\right)+12\left(170\right)+12\left(128\right)+6\left(186\right)\\ +2\left(232\right)+6\left(128\right)+\left(3n-6\right)\left(144\right)+12\left(114\right)+\left(12n-42\right)\left(116\right)\\ +\left(12n-36\right)\left(130\right)+\left(6n-18\right)\left(176\right)+\left(6n-18\right)\left(190\right)\\ +6\left(132\right)+\left(n-4\right)\left(236\right)=4792+5816n. \end{array}$$
(2) The neighborhood second *Zagreb* index of **HCP**(**n**) is defined as follows:
  (20) $$\begin{array}{c} M_{2}^{*}\left(G\right)=\sum_{st\in E\left(G\right)}\left(\delta_{s}\delta_{t}\right)=\sum_{st\in E_{1}}\left(\delta_{s}\delta_{t}\right)+\sum_{st\in E_{2}}\left(\delta_{s}\delta_{t}\right)+\cdots +\\ \sum_{st\in E_{21}}\left(\delta_{s}\delta_{t}\right)=12\left(1225\right)+12\left(2380\right)12\left(2240\right)\\ +12\left(1960\right)2\left(7752\right)+12\left(3136\right)+12\left(3584\right)\\ +6\left(7269\right)+12\left(6384\right)+12\left(4032\right)+6\left(8208\right)+2\left(13452\right)+6\left(4096\right)\\ +\left(3n-6\right)\left(5184\right)+12\left(3248\right)+\left(12n-42\right)\left(3364\right)\\ +\left(12n-36\right)\left(4276\right)+\left(6n-18\right)\left(6844\right)+\left(6n-18\right)\left(8496\right)\\ +6\left(4352\right)+\left(n-4\right)\left(13924\right)=211996n-130156. \end{array}$$
(3) The neighborhood forgotten topological index of **HCP**(**n**) is defined as follows:
  (21) $$\begin{array}{c} F_{N}^{*}\left(G\right)=\sum_{st\in E\left(G\right)}\left(\delta_{s}^{2}\delta_{t}^{2}\right)=\sum_{st\in E_{1}}\left(\delta_{s}^{2}\delta_{t}^{2}\right)+\sum_{st\in E_{2}}\left(\delta_{s}^{2}\delta_{t}^{2}\right)+\sum_{st\in E_{3}}\left(\delta_{s}^{2}\delta_{t}^{2}\right)+\cdots +\sum_{st\in E_{21}}\left(\delta_{s}^{2}\delta_{t}^{2}\right)\\ =12\left(2450\right)+12\left(5849\right)\\ +12\left(5321\right)+12\left(4361\right)+2\left(17620\right)+12\left(6272\right)\\ +12\left(7232\right)+6\left(17092\right)+12\left(16132\right)+12\left(8320\right)\\ +6\left(18180\right)+2\left(26920\right)\\ +6\left(8192\right)+\left(3n-6\right)\left(10368\right)+12\left(6500\right)+\left(12n-42\right)\left(6728\right)\\ +\left(12n-36\right)\left(8548\right)+\left(6n-18\right)\left(17288\right)+\left(6n-18\right)\\ \left(19108\right)+6\left(8720\right)+\left(n-4\right)\left(27848\right)=460640n-267604. \end{array}$$
(4) The neighborhood second modified *Zagreb* index of **HCP**(**n**) is defined as follows:
  (22) $$\begin{array}{c} M_{2}^{nm}\left(G\right)=\sum_{st\in E\left(G\right)}\frac{1}{\delta_{s}\delta_{t}}+\sum_{st\in E_{2}}\frac{1}{\delta_{s}\delta_{t}}+\sum_{st\in E_{3}}\frac{1}{\delta_{s}\delta_{t}}+\cdots +\sum_{st\in E_{21}}\frac{1}{\delta_{s}\delta_{t}}\\ =129\left(\frac{1}{1225}\right)+12\left(\frac{1}{2380}\right)+12\left(\frac{1}{2240}\right)\\ +12\left(\frac{1}{1960}\right)+2\left(\frac{1}{7752}\right)+12\left(\frac{1}{3136}\right)+12\left(\frac{1}{3584}\right)\\ +6\left(\frac{1}{7296}\right)+12\left(\frac{1}{6384}\right)+12\left(\frac{1}{4032}\right)+6\left(\frac{1}{8208}\right)+2\left(\frac{1}{13452}\right)+6\left(\frac{1}{4096}\right)\\ +\left(3n-6\right)\left(\frac{1}{5184}\right)+12\left(\frac{1}{3248}\right)+\left(12n-42\right)\left(\frac{1}{3364}\right)\\ +\left(12n-36\right)\left(\frac{1}{4176}\right)+\left(6n-18\right)\left(\frac{1}{6844}\right)+\left(6n-18\right)\left(\frac{1}{8496}\right)+6\left(\frac{1}{4352}\right)\\ +\left(n-4\right)\left(\frac{1}{13924}\right)=211996n-130156. \end{array}$$
(5) The third and fifth NDe index of **HCP**(**n**) is defined as follows:
  (23) $$\begin{array}{c} ND_{3}\left(G\right)=\sum_{st\in E\left(G\right)}\delta_{s}\delta_{t}\left(\delta_{s}+\delta_{t}\right)==\sum_{st\in E_{1}}\delta_{s}\delta_{t}\left(\delta_{s}+\delta_{t}\right)+\sum_{st\in E_{2}}\delta_{s}\delta_{t}\left(\delta_{s}+\delta_{t}\right)+\sum_{st\in E_{3}}\delta_{s}\delta_{t}\left(\delta_{s}+\delta_{t}\right)+\cdots +\sum_{st\in E_{21}}\delta_{s}\delta_{t}\left(\delta_{s}+\delta_{t}\right)\\ =12\left(1225\right)\left(70\right)+12\left(2380\right)\left(103\right)+12\left(2240\right)\left(99\right)+12\left(1960\right)\left(91\right)+2\left(7752\right)\left(182\right)+12\left(3136\right)\left(112\right)+12\left(3684\right)\left(120\right)\\ +6\left(7296\right)\left(178\right)+12\left(6384\right)\left(677\right)+12\left(4032\right)\left(128\right)+6\left(8208\right)\left(186\right)+2\left(13452\right)\left(232\right)+6\left(4096\right)\left(128\right)+\left(3n-6\right)\left(5184\right)\left(144\right)\\ +12\left(3248\right)\left(114\right)+\left(12n-42\right)\left(3364\right)\left(116\right)+\left(12n-36\right)\left(4176\right)\left(130\right)+\left(6n-18\right)\left(6844\right)\left(176\right)+\left(6n-18\right)\left(8496\right)\left(190\right)\\ +6\left(4352\right)\left(132\right)+\left(n-4\right)\left(13924\right)\left(236\right)=33635504n+8961688,\\ ND_{5}\left(G\right)=\sum_{st\in E\left(G\right)}\left[\frac{\delta_{s}}{\delta_{t}}+\frac{\delta_{t}}{\delta_{s}}\right]==\sum_{st\in E_{1}}\left[\frac{\delta_{s}}{\delta_{t}}+\frac{\delta_{t}}{\delta_{s}}\right]+\sum_{st\in E_{2}}\left[\frac{\delta_{s}}{\delta_{t}}+\frac{\delta_{t}}{\delta_{s}}\right]\\ +\sum_{st\in E_{3}}\left[\frac{\delta_{s}}{\delta_{t}}+\frac{\delta_{t}}{\delta_{s}}\right]+\cdots +\sum_{st\in E_{21}}\left[\frac{\delta_{s}}{\delta_{t}}+\frac{\delta_{t}}{\delta_{s}}\right]=12\left(2\right)+12\left(2.457563025\right)\\ +12\left(\frac{5321}{2240}\right)+12\left(\frac{89}{40}\right)+2\left(\frac{4405}{1938}\right)+12\left(2\right)\\ +12\left(\frac{113}{56}\right)+6\left(\frac{4273}{1824}\right)+12\left(\frac{4033}{1596}\right)\\ +12\left(\frac{130}{63}\right)+6\left(\frac{505}{228}\right)+2\left(\frac{6730}{3363}\right)+6\left(2\right)\\ +\left(3n-6\right)\left(2\right)+12\left(\frac{1625}{812}\right)+\left(12n-42\right)\left(2\right)+\left(12n-36\right)\left(\frac{2137}{1044}\right)+\left(6n-18\right)\left(\frac{4322}{1711}\right)+\left(6n-18\right)\left(\frac{4777}{2124}\right)+6\left(\frac{545}{272}\right)\\ +n-42=32.28529324+85.21361777n. \end{array}$$
(6) The neighborhood Harmonic index of **HCP**(**n**) is defined as follows:
  (24) $$\begin{array}{c} NH\left(G\right)=\sum_{st\in E\left(G\right)}\frac{2}{\delta_{s}+\delta_{t}}=\sum_{st\in E_{1}}\frac{2}{\delta_{s}+\delta_{t}}+\sum_{st\in E_{2}}\frac{2}{\delta_{s}+\delta_{t}}+\sum_{st\in E_{3}}\frac{2}{\delta_{s}+\delta_{t}}+\cdots +\sum_{st\in E_{21}}\frac{2}{\delta_{s}+\delta_{t}}\\ +12\left(\frac{1}{35}\right)+12\left(\frac{2}{103}\right)+12\left(\frac{2}{99}\right)\\ +12\left(\frac{2}{91}\right)+2\left(\frac{1}{91}\right)+12\left(\frac{1}{56}\right)+12\left(\frac{1}{60}\right)+6\left(\frac{1}{89}\right)\\ +12\left(\frac{1}{85}\right)+12\left(\frac{1}{64}\right)+6\left(\frac{1}{93}\right)+2\left(\frac{1}{116}\right)+6\left(\frac{1}{64}\right)+\left(3n-6\right)\left(\frac{1}{72}\right)+12\left(\frac{1}{57}\right)\\ +\left(12n-42\right)\left(\frac{1}{58}\right)+\left(12n-36\right)\left(\frac{1}{65}\right)\\ +\left(6n-18\right)\left(\frac{1}{88}\right)+\left(6n-18\right)\left(\frac{1}{95}\right)+6\left(\frac{1}{66}\right)\\ +\left(n-4\right)\left(\frac{1}{118}\right)=0.5729928922n+0.6020913479. \end{array}$$
(7) The neighborhood inverse sum index of **HCP**(**n**) is defined as follows:
  (25) $$\begin{array}{c} NI\left(G\right)=\sum_{st\in E\left(G\right)}\frac{\delta_{s}\delta_{t}}{\delta_{s}+\delta_{t}}=\sum_{st\in E_{1}}\frac{\delta_{s}\delta_{t}}{\delta_{s}+\delta_{t}}+\sum_{st\in E_{2}}\frac{\delta_{s}\delta_{t}}{\delta_{s}+\delta_{t}}+\sum_{st\in E_{3}}\frac{\delta_{s}\delta_{t}}{\delta_{s}+\delta_{t}}+\cdots \\ +\sum_{st\in E_{21}}\frac{\delta_{s}\delta_{t}}{\delta_{s}+\delta_{t}}=12\left(\frac{35}{2}\right)+12\left(\frac{2380}{103}\right)+12\left(\frac{2240}{99}\right)\\ +12\left(\frac{280}{13}\right)+2\left(\frac{3876}{91}\right)+12\left(28\right)+12\left(\frac{448}{15}\right)+6\left(\frac{3648}{89}\right)\\ +12\left(\frac{3192}{85}\right)+12\left(\frac{63}{2}\right)+6\left(\frac{1368}{31}\right)+2\left(\frac{3363}{58}\right)+6\left(32\right)+\left(3n-6\right)\left(36\right)\\ +12\left(\frac{1624}{57}\right)+\left(12n-42\right)\left(29\right)+\left(12n-36\right)\left(\frac{2088}{65}\right)\\ +\left(6n-18\right)\left(\frac{1711}{44}\right)+\left(6n-18\right)\left(\frac{4248}{95}\right)+6\left(\frac{1088}{33}\right)\\ +\left(n-4\right)\left(59\right)=1402.089842n-347.4039608. \end{array}$$
(8) The Sanskruti index of **HCP**(**n**) is defined as follows:
  (26) $$\begin{array}{c} NI\left(G\right)=\sum_{st\in E\left(G\right)}{\left(\frac{\delta_{s}\delta_{t}}{\delta_{s}+\delta_{t}-2}\right)}^{3}=\sum_{st\in E_{1}}{\left(\frac{\delta_{s}\delta_{t}}{\delta_{s}+\delta_{t}-2}\right)}^{3}+\sum_{st\in E_{2}}{\left(\frac{\delta_{s}\delta_{t}}{\delta_{s}+\delta_{t}-2}\right)}^{3}+\sum_{st\in E_{3}}{\left(\frac{\delta_{s}\delta_{t}}{\delta_{s}+\delta_{t}-2}\right)}^{3}+\cdots +\sum_{st\in E_{21}}{\left(\frac{\delta_{s}\delta_{t}}{\delta_{s}+\delta_{t}-2}\right)}^{3}\\ =12{\left(\frac{35\times 35}{35+35-2}\right)}^{3}+12{\left(\frac{35\times 68}{35+68-2}\right)}^{3}+12{\left(\frac{35\times 64}{35+64-2}\right)}^{3}\\ +12{\left(\frac{35\times 56}{35+56-2}\right)}^{3}+2{\left(\frac{68\times 114}{68+114-2}\right)}^{3}+12{\left(\frac{56\times 56}{56+56-2}\right)}^{3}\\ +12{\left(\frac{56\times 64}{56+64-2}\right)}^{3}+6{\left(\frac{64\times 114}{64+114-2}\right)}^{3}+12{\left(\frac{56\times 114}{56+114-2}\right)}^{3}\\ +12{\left(\frac{56\times 72}{56+72-2}\right)}^{3}+6{\left(\frac{114\times 72}{114+72-2}\right)}^{3}+2{\left(\frac{114\times 118}{114+118-2}\right)}^{3}\\ +6{\left(\frac{64\times 64}{64+64-2}\right)}^{3}+\left(3n-6\right){\left(\frac{72\times 72}{72+72-2}\right)}^{3}+12{\left(\frac{58\times 56}{58+56-2}\right)}^{3}\\ +\left(12n-42\right){\left(\frac{58\times 58}{58+58-2}\right)}^{3}+\left(12n-36\right){\left(\frac{58\times 72}{58+72-2}\right)}^{3}\\ +\left(6n-18\right){\left(\frac{58\times 118}{58+118-2}\right)}^{3}+\left(6n-18\right){\left(\frac{118\times 72}{118+72-2}\right)}^{3}\\ +6{\left(\frac{68\times 64}{68+64-2}\right)}^{3}\left(n-4\right){\left(\frac{118\times 118}{118+118-2}\right)}^{3}=2000631.573n-1847249.828. \end{array}$$

## 4. Conclusion

From Table 5, we can exactly say that all the descriptors behave positively except exponential reduced Zagreb index and shown in Table 5 by symbol (+) and(−) as the value *n* increases and the descriptor *ND*_3_ dominates amongst all the topological descriptors which assure us about most dominant topological property for HCP(*n*), hexagonal closed-packed crystal structure lattice. We compute all the above calculated descriptors for *n*=1, *i.e.,* for a unit hexagonal close-packed crystal cell.

## Figures and Tables

**Table 1:** Topological indices and their corresponding functions.

Founders	Name	Defining H and K	*TI*=*f*(*H*, *K*)
Gutman [10]	FirstZagreb	*H*=*d*_*s*_ and *K*=*d*_*t*_	*M*_1_(*G*)=(*H*+*K*)
	Second Zagreb		*M*_2_(*G*)=(*H* × *K*)
Bollobás et al. and Amić et al. [11, 12]	General Randić index		*R*_*α*_(*G*)=(*H* × *K*)^*α*^
Estrada et al. [13]	Atom bond connectivity index		$ABC\left(G\right)=\sqrt{H+K-2/H\times K}$
Vukičević et al. [14]	Geometric arithmetic index		$GA\left(G\right)=2\sqrt{H\times K}/H+K$
Chen et al. [15]	Exponential reduced Zagreb index		*e* ^*RM*2^(*G*)=*e*^(*H* − 1)(*K* − 1)^
Zhao et al. [16]	General Reverse Randić index,where *α* ∈ *R*	*H*=ℜ_*s*_ and *K*=ℜ_*t*_	*R*_*α*_(*G*)=[*H* × *K*]^*α*^
Estrada et al. [13]	Reverse Atom Bond Connectivity index		$\mathbb{R}ABC\left(G\right)=\sqrt{H+K-2/H\times K}$
Vukičević et al. [13]	Reverse geometric arithmetic index		$\mathbb{R}GA\left(G\right)=2\sqrt{H\times K}/H+K$
Shirdel et al. [17]	Reverse hyper Zagreb index		ℝ*HM*(*G*)=(*H*+*K*)^2^
Ghorbani et al. [18]	Neighborhood versio of first Zagreb index	*H*=*δ*_*s*_ and *K*=*δ*_*t*_	*M*_1_^*∗*^(*G*)=(*H*+*K*)
Monadal [19]	Neighborhood second Zagreb index		*M*_2_^*∗*^(*G*)=(*H* × *K*)
	Neighborhood Forgotten index		*F*_*N*_^*∗*^(*G*)=(*H*^2^+*K*^2^)
Siddiqui et al. [20]	Neighborhood second modified Zagreb index		*M*_2_^*nm*^(*G*)=1/*H* × *K*
Pal et al. [21]	Third *N* *De*		*ND*_3_=*HK*(*H*+*K*)
	Fifth *N* *De*		*ND*_5_=[*H*/*K*+*K*/*H*]
	Neighborhood Harmonic index		*NH*(*G*)=2/*H*+*K*
	Neighborhood Inverse sum index		*NI*(*G*)=*H* × *K*/*H*+*K*
Hosamani et al. [22]	The Sanskruti index		*S*(*G*)=(*H* × *K*/*H*+*K*−2)^3^

**Table 2:** Edge partition of *HCP*(*n*) based on degree of *V*(HCP(*n*))

Edge representation	(**d**_**s**_, **d**_**t**_)	Cardinality **of** (**d**_**s**_, **d**_**t**_)
**E**_1_	(5,5)	12
**E**_2_	(5,10)	12
**E**_3_	(5,8)	12
**E**_4_	(8,8)	3*n*
**E**_5_	(8,10)	6
**E**_6_	(7,7)	12*n* − 18
**E**_7_	(7,8)	12*n* − 12
**E**_8_	(7,14)	6*n* − 6
**E**_9_	(10,14)	2
**E**_10_	(14,14)	*n* − 2
**E**_11_	(8,14)	6*n* − 6
**E**_12_	(5,7)	12

**Table 3:** Edge partition of HCP(*n*) based on reverse degree of *V*(HCP(*n*))

Edge representation	(ℝ_**s**_, ℝ_**t**_)	Cardinality of (*ℝ*_*s*_, *ℝ*_*t*_)
**E**_1_	(10,10)	12
**E**_2_	(10,5)	12
**E**_3_	(10,7)	12
**E**_4_	(7,7)	3*n*
**E**_5_	(7,5)	6
**E**_6_	(8,8)	12*n* − 18
**E**_7_	(8,7)	12*n* − 12
**E**_8_	(8,1)	6*n* − 6
**E**_9_	(5,1)	2
**E**_10_	(1,1)	*n* − 2
**E**_11_	(7,1)	6*n* − 6
**E**_12_	(10,8)	12

**Table 4:** Edge partition of HCP(*n*) based on neighborhood degree of *V*(HCP(*n*))

Edge representation	(**N**_**s**_,**N**_**t**_)	Cardinality of(**N**_**s**_, **N**_**t**_)
**E**_1_	(35,35)	12
**E**_2_	(35,68)	12
**E**_3_	(35,64)	12
**E**_4_	(35,56)	12
**E**_5_	(68,114)	2
**E**_6_	(56,56)	12
**E**_7_	(56,64)	12
**E**_8_	(64,114)	6
**E**_9_	(56,114)	12
**E**_10_	(56,72)	12
**E**_11_	(114,72)	6
**E**_12_	(114,118)	2
**E**_13_	(64,64)	6
**E**_14_	(72,72)	3*n* − 6
**E**_15_	(58,56)	12
**E**_16_	(58,58)	12*n* − 42
**E**_17_	(58,72)	12*n* − 36
**E**_18_	(58,118)	6*n* − 18
**E**_19_	(118,72)	6*n* − 18
**E**_20_	(68,64)	6
**E**_21_	(118,118)	*n* − 4

**Table 5:** Analyzing descriptors.

Descriptors	General closed formula for hexagonal crystal structure lattice	*n*=1=one unit cell	Check for positivity (+),negativity(−) , and domination
**M**_1_(**HCP**(**n**))	643*n* − 10	**633**	(+)
**R**_1_(**HCP**(**n**))=**M**_2_(**HCP**(**n**))	2908*n* − 646	**2262**	(+)
**R**_−1_(**HCP**(**n**))	0.579*n*+0.7455	**1.3245**	(+)
**R**_1/2_(**HCP**(**n**))	334.69*n*+2.38	**337.07**	(+)
**R**_−1/2_(**HCP**(**n**))	4.94*n*+3.37	**8.31**	(+)
**ABC**(**HCP**(**n**))	18.66*n*+8.495	**27.155**	(+)
**GA**(**HCP**(**n**))	39.4027*n*+11.354	**50.7567**	(+)
**e** ^**RM**2^(**G**)	2.4875 × 10^73^*n* − 4.975 × 10^73^	**-2.487524929×10** ^**73**^	(−)
ℝ**R**_1_(**HCP**(**n**))	1678*n*+1904	**3582**	(+)
ℝ**R**_−1_(**HCP**(**n**))	2407/784*n* − 15959/5600	**0.2203316**	(+)
ℝ**R**_1/2_(**HCP**(**n**))	240.6448*n*+183.907	**424.5518**	(+)
ℝ**R**_−1/2_(**HCP**(**n**))	8.92*n* − 3.50	**5.42**	(+)
ℝ**ABC**(**HCP**(**n**))	24.04*n*+1.7595	**25.7995**	(+)
ℝ**GA**(**HCP**(**n**))	35.71*n*+14.7447	**50.4547**	(+)
ℝ**HM**(**HCP**(**n**))	7234*n*+7606	**14840**	(+)
**M**_1_^**∗**^(**HCP**(**n**))	4792+5816*n*	**10608**	(+)
**M**_2_^**∗**^(**HCP**(**n**))	211996*n* − 130156	**81840**	(+)
**M**_*N*_^**∗**^(**HCP**(**n**))	460640*n* − 267604	**193036**	(+)
**M**_2_^**nm**^(**HCP**(**n**))	211996*n* − 130156	**81840**	(+)
**N** **D**_3_(**HCP**(**n**))	33635504*n*+8961688 42597192	**42597192**	(+) and dominates
**N** **D**_5_(**HCP**(**n**))	32.28529324+85.21361777*n*	**117.4989**	(+)
**NH**(**HCP**(**n**))	0.5729928922*n*+0.6020913479	**1.17508424**	(+)
**NI**(**HCP**(**n**))	1402.089842*n* − 347.4039608	**1054.685881**	(+)
**S**(**HCP**(**n**))	2000631.573*n* − 1847249.828	**153381.745**	(+)

## Data Availability

The data used to support the findings of this study are cited at relevant places within the articles references.
